# The L-Cysteine Desulfurase NFS1 Is Localized in the Cytosol where it Provides the Sulfur for Molybdenum Cofactor Biosynthesis in Humans

**DOI:** 10.1371/journal.pone.0060869

**Published:** 2013-04-12

**Authors:** Zvonimir Marelja, Mita Mullick Chowdhury, Carsten Dosche, Carsten Hille, Otto Baumann, Hans-Gerd Löhmannsröben, Silke Leimkühler

**Affiliations:** 1 Department of Molecular Enzymology, Institute of Biochemistry, University of Potsdam, Potsdam, Germany; 2 Department of Physical Chemistry, Institute of Chemistry, University of Potsdam, Potsdam, Germany; 3 Department of Animal Physiology, Institute of Biochemistry, University of Potsdam, Potsdam, Germany; Queen Mary University of London, United Kingdom

## Abstract

In humans, the L-cysteine desulfurase NFS1 plays a crucial role in the mitochondrial iron-sulfur cluster biosynthesis and in the thiomodification of mitochondrial and cytosolic tRNAs. We have previously demonstrated that purified NFS1 is able to transfer sulfur to the C-terminal domain of MOCS3, a cytosolic protein involved in molybdenum cofactor biosynthesis and tRNA thiolation. However, no direct evidence existed so far for the interaction of NFS1 and MOCS3 in the cytosol of human cells. Here, we present direct data to show the interaction of NFS1 and MOCS3 in the cytosol of human cells using Förster resonance energy transfer and a split-EGFP system. The colocalization of NFS1 and MOCS3 in the cytosol was confirmed by immunodetection of fractionated cells and localization studies using confocal fluorescence microscopy. Purified NFS1 was used to reconstitute the lacking molybdoenzyme activity of the *Neurospora crassa nit-1* mutant, giving additional evidence that NFS1 is the sulfur donor for Moco biosynthesis in eukaryotes in general.

## Introduction

L-cysteine desulfurases are pyridoxal phosphate (PLP)-dependent enzymes that use L-cysteine as substrate to produce L-alanine and a protein-bound persulfide [1], [2]. In humans, the L-cysteine desulfurase NFS1 is the orthologue of *Azotobacter vinelandii* NifS and *Escherichia coli* IscS [1], [3]. NFS1 is mainly localized in mitochondria where it acts as a component of the mitochondrial iron-sulfur (FeS) cluster (ISC) assembly machinery, required for the maturation of mitochondrial, cytosolic and nuclear FeS proteins [4], [5], [6]. Within the mitochondria, NFS1 forms a complex with ISD11, a 10 kDa protein that functions as stabilizer of NFS1 [7], [8]. While L-cysteine desulfurases are highly conserved throughout all kingdoms of life, ISD11 is only found in eukaryotes, suggesting a unique and novel function of the protein for this class of organisms [9]. NFS1/ISD11 additionally binds to the scaffold protein ISCU forming the ternary ISCU/NFS1/ISD11 complex [10]. This complex provides the platform for binding of frataxin, a protein of the core ISC assembly machinery regulating the activity of the ternary complex [11]. Since binding sites for iron have been detected on its surface, frataxin has also been proposed to be the iron donor for the ISC assembly machinery in former studies, however, this role is not clear [12], [13], [14].

Maturation of extramitochondrial FeS proteins requires the assistance of the cytosolic FeS assembly (CIA) machinery [6]. It has been suggested that the core mitochondrial ISC assembly machinery synthesizes a sulfur-containing component that is exported to the cytosol and utilized by the CIA machinery [6]. Recent studies suggested the involvement of an ABC transporter of the mitochondrial inner membrane (Atm1 in yeast, ABCB7 in higher eukaryotes), since depletion of this ABC transporter or components of the core ISC assembly machinery resulted in the impairment of the maturation of extramitochondrial FeS proteins and mitochondrial iron overload [15], [16]. However, the nature of the transported sulfur-compound remains unresolved so far.

A link between FeS cluster biogenesis and 2-thiouridine (s^2^U) modification of tRNAs was identified in *E. coli* and *Saccharomyces cerevisiae*. In yeast, tRNA thiolation occurs in both the cytosol and mitochondria [17]. Additionally, NFS1 was also shown to be involved in the thiolation of cytosolic tRNAs [17], [18]. Further studies in human cells and yeast showed an additional localization of NFS1 and ISD11 in the nucleus [19], [20], [21], however, the role of NFS1 and ISD11 in the nucleus is not clear to date [18] **.**

For 5-methoxycarbonylmethyl-2-thiouridine (mcm^5^s^2^U_34_) modification of cytosolic tRNAs, sulfur is transferred from the rhodanese-like protein MOCS3 (Uba4 in yeast) to the C-terminus of the ubiquitin-related modifier URM1 (Urm1 in yeast) [22], [23], [24], [25], [26]. The N-terminal domain of MOCS3 shares amino acid sequence homologies to ubiquitin-activating E1-like enzymes and to the *E. coli* MoeB protein [27]. In the activated MOCS3-URM1 acyl-adenylate complex, the persulfide sulfur is transferred from the C-terminal MOCS3 rhodanese-like domain (RLD) to URM1, forming a thiocarboxylate group at C-terminal Gly of URM1 and releasing MOCS3 and AMP [22], [26].

In addition, human MOCS3 was initially identified to be involved in molybdenum cofactor (Moco) biosynthesis in the cytosol [27]. Here, MOCS3 interacts with MOCS2A and also forms a thiocarboxylate group at the C-terminus of MOCS2A [22], [26], [27]. MOCS2A subsequently assembles with MOCS2B to form the molybdopterin (MPT) synthase [28]. The MPT synthase binds the first intermediate of Moco biosynthesis, cyclic pyranopterin monophosphate (cPMP) and generates MPT after the transfer of two sulfur atoms from two MOCS2A proteins [28]. MOCS2B binds cPMP in this reaction. The two sulfur atoms of MPT coordinate the molybdenum atom in the final step of Moco biosynthesis. In humans, Moco is required for the activity of xanthine dehydrogenase, aldehyde oxidase, sulfite oxidase and the mitochondrial amidoxime reducing components, mARC1 and mARC2 [29].

Previously we showed that the sulfur from purified MOCS3 can be further transferred to MOCS2A and URM1 [26]. We also suggested that a cysteine desulfurase might be the sulfur donor for MOCS3 [27], [30], [31]. By *in vitro* studies using purified NFS1 and MOCS3-RLD, it was demonstrated that the sulfur is mobilized from L-cysteine by NFS1 forming a persulfide group on its conserved Cys381 which is further transferred to Cys412 of MOCS3-RLD [31].

In order to confirm the cytosolic role of NFS1 for Moco biosynthesis *in vivo*, we showed the cytosolic localization of NFS1 and performed interaction studies between NFS1 and MOCS3 in HeLa cells. The ability of NFS1 to act as sulfur donor for MPT formation was analyzed by a reconstitution assay using purified proteins and cell lysates of the *Neurospora crassa nit-1* mutant that is known to lack molybdoenzyme activity. Our studies provide evidence for the presence of NFS1 in the cytosol and a role in Moco biosynthesis.

## Results

### Immunodetection of NFS1 and MOCS3

**Figure 1:**
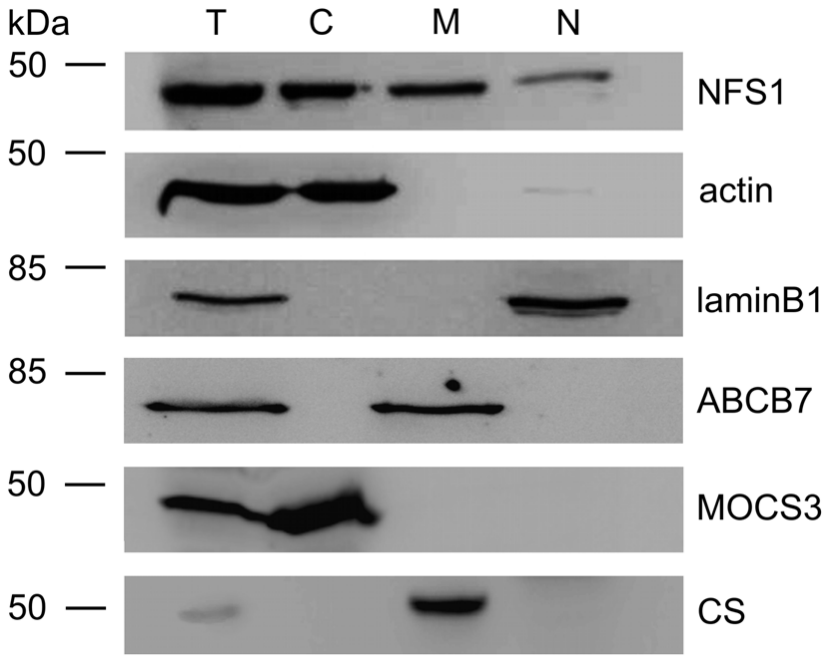
Immunodetection of NFS1 and MOCS3 after subcellular fractionation of HeLa cells. Total protein extracts (T), cytosol (C), mitochondria (M), and nucleus (N) were prepared separately from 80–90% confluent HeLa cells to avoid cross contaminations of the compartments. Proteins of each cellular fraction were analyzed by immunoblotting using the following antibodies: anti-NFS1 (*top panel*), anti-γ-actin as cytosolic marker control (*second panel*), anti-laminB1 as nuclear marker (*third panel*), anti-ABCB7 as mitochondrial inner membrane marker (*fourth panel*), anti-MOCS3 as cytosolic marker (*fifth panel*), and anti-citrate synthase [6] as mitochondrial matrix marker (*bottom*).

To analyze the subcellular localization of NFS1, we performed immunodetection analyses in subcellular fractions of HeLa cells using NFS1 antibodies. HeLa cells were grown until 80% confluency, gently harvested, lysed and fractionated into cytosolic, mitochondria and nucleus fractions. The cytosolic fraction was at least 32-fold concentrated. The results in Figure 1 show that we were able to detect NFS1 in the mitochondria and nucleus, as reported previously [4], [5], [19], [20]. However, we were also able to detect NFS1 in the cytosolic fraction [5], [32]. As control for the cytosolic fraction, and to confirm the purity of the fractions, we used MOCS3 and γ-actin antibodies. While MOCS3 was detected in the cytosol [26], [27], γ-actin was additionally detected in low amounts in the nucleus fraction, due to its association with the nuclear membrane. Additionally, the quality of the subcellular fractionation was analyzed by using markers for the mitochondrial inner membrane transporter ABCB7, the mitochondrial matrix protein citrate synthase, and the nuclear protein laminB1. Here, ABCB7 and citrate synthase were only detected in the mitochondrial fraction and laminB1 in the nuclear fraction (Figure 1). Thus, these data provide strong evidence for the localization of NFS1 in the cytosol of HeLa cells.

### Fluorescent Microscopy of EYFP/ECFP Fusion Proteins Expressed in HeLa Cells

**Figure 2:**
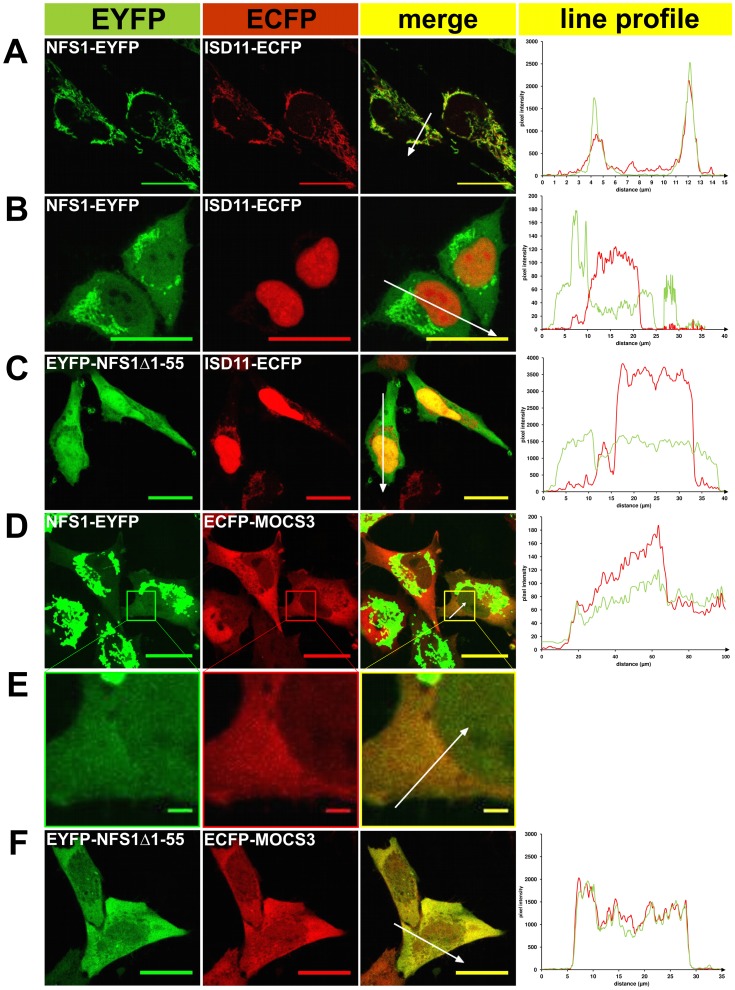
Fluorescent microscopy of EYFP/ECFP fusion proteins expressed in HeLa cells. EYFP (*green pseudocolor*) and ECFP-tagged (*red pseudocolor*) proteins were analyzed in HeLa cells for subcellular localization and co-localization (the “*merge*” row resulted in a *yellow color* when colocalization occurred) by fluorescence confocal microscopy. In addition, a line profile plot (*right*) shows the pixel intensities of EYFP and ECFP along an arrow (distance in µm) presented in the “merge” figure. *A,* NFS1-EYFP and ISD11-ECFP (mitochondrial localization); *B*, NFS1-EYFP (cytosolic localization) and ISD11-ECFP (nuclear localization); *C*, EYFP-NFS1Δ1-55 and ISD11-ECFP; *D*, *E* NFS1-EYFP and ECFP-MOCS3; *F*, EYFP-NFS1Δ1-55 and ECFP-MOCS3. The figures of *panel E* are a *close-up* figure of an indicated area (box) in the figures of *panel D*. Scale bars, 20 µm (except *E,* which is 2 µm).

**Figure 3:**
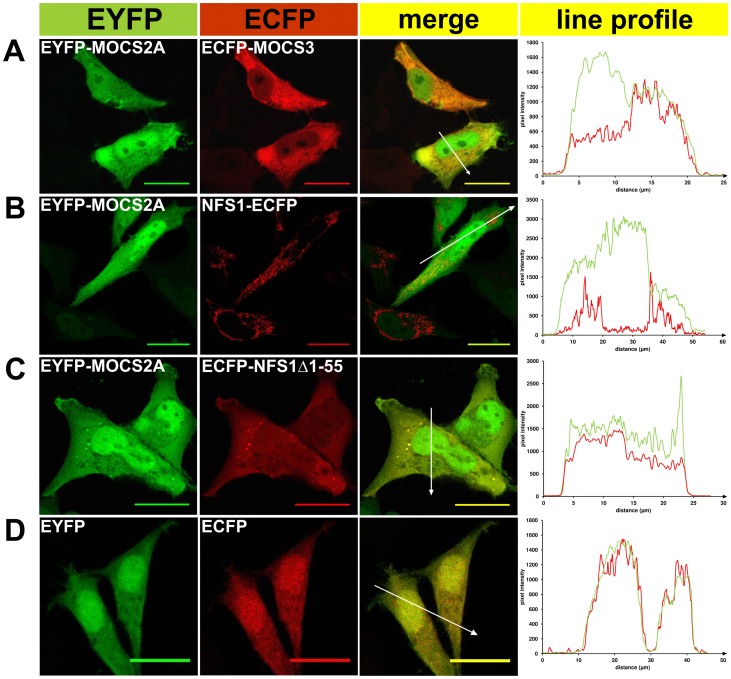
Fluorescent microscopy of EYFP/ECFP fusion proteins expressed in HeLa cells. EYFP (*green pseudocolor*) and ECFP-tagged (*red pseudocolor*) proteins were analyzed in HeLa cells for subcellular localization and co-localization (the “*merge*” row resulted in a *yellow color* when colocalization occured) by fluorescence confocal microscopy. In addition, a line profile plot (*right*) shows the pixel intensities of EYFP and ECFP along an arrow (distance in µm) presented in the “merge” figure. *A,* EYFP-MOCS2A and ECFP-MOCS3; *B*, EYFP-MOCS2A and NFS1-ECFP; *C*, EYFP-MOCS2A and ECFP-NFS1Δ1-55; *D*, EYFP and ECFP. Scale bars, 20 µm.

To analyze the subcellular localization of NFS1 in HeLa cells we constructed N-terminal and C-terminal fusion proteins to ECFP and EYFP. For colocalization of NFS1-EYFP or EYFP-NFS1Δ1-55 with either ISD11-ECFP or ECFP-MOCS3 the corresponding pairs of fusion proteins were transiently coexpressed in HeLa cells and subcellular localization was visualized by confocal fluorescent microscopy as shown in Figure 2 and 3. Colocalization of proteins was indicated by both merging the EYFP and ECFP fluorescence, resulting in a yellow color (see “merge” row in Figure 2 and 3), and by the line profile, comparing the pixel intensities of EYFP and ECFP along the indicated arrow in the merged images (see right row in Figure 2 and 3). As controls ISD11, MOCS2A and MOCS3 were fused to ECFP or EYFP. Additionally, we analyzed the localization of ISD11, the partner protein of NFS1 in mitochondria.

As shown in Figure 2A, in the cells expressing both NFS1-EYFP and ISD11-ECFP, the majority of NFS1-EYFP and ISD11-ECFP were targeted to the mitochondria, where both proteins were colocalized. The localization of both proteins in the mitochondria was confirmed with Mitotracker and is shown as supplemental Figure S1 which is published as supplemental data on the PloS ONE web site. In general, when the microscopic gain was increased for both transfected and untransfected cells, fluorescence in the cytosol was only visible for transfected cells expressing NFS1-EYFP (Figure 2 D and E, data for untransfected cells are not shown). Interestingly, without increasing the gain only a small fraction of fluorescent cells (24%, N = 140), mainly neighboring cells, showed targeting of NFS1-EYFP to the cytosol (Figure 2B).

In contrast, a fluorescence of ISD11-ECFP was not detected in the cytosol, indicating either a low abundance or its absence in this compartment. Since ISD11 and NFS1 were shown to colocalize in mitochondria and ISD11 and NFS1Δ1-55 form a complex *in vitro* [31], we also expected a colocalization in the cytosol. Thus, to analyze this further and to increase the concentration of NFS1 in the cytosol we cleaved off its mitochondrial targeting signal. We cotransfected cells with EYFP-NFS1Δ1-55 and ISD11-ECFP and analyzed the effect of the localization of ISD11. As expected, EYFP-NFS1Δ1-55 was mainly detected in the cytosol. In contrast, ISD11-ECFP was solely detected in the mitochondria and in the nucleus (Figure 2C). In addition, NFS1-EYFP and EYFP-NFS1Δ1-55 were showing a localization in the nucleus (Figure 2B and C). This localization of NFS1 and ISD11 has been reported previously by immunodetection [5], [21], [33].

Additionally, we analyzed the localization of ECFP-MOCS3 with either NFS1-EYFP or EYFP-NFS1Δ1-55 after cotransfection (Figure 2D–F). Consistent with the colocalizations shown above, NFS1-EYFP was predominantly localized in the mitochondria but also showed localization in the cytosol and nucleus. This shows that the cotransfected protein does not influence the localization of NFS1. The localization of ECFP-MOCS3 was mainly detected in the cytosol. When the cytosolic form EYFP-NFS1Δ1-55 and ECFP-MOCS3 were cotransfected, the colocalization signal in the cytosol was increased (Figure 2F). As control, we demonstrated that EYFP-MOCS2A and ECFP-MOCS3 had a colocalization in the cytosol, while MOCS2A was also targeted to the nucleus (Figure 3), as reported previously [26], [27].

In total, these observations show that NFS1 and ISD11 are present in mitochondria and in the nucleus, while NFS1 is additionally localized in the cytosol where we also detected MOCS3.

### Detection of *in vivo* Protein-protein Interactions Using the Split-EGFP System

To analyze the direct interaction of NFS1 and MOCS3 in the cytosol, we used a split enhanced green fluorescence protein (split-EGFP) complementation assay. For this purpose we fused NFS1, NFS1Δ1-55, ISD11 and MOCS3 to the N-terminal 1–157 amino acids or the C-terminal 158–238 amino acids of EGFP and cotransfected the corresponding plasmids in HeLa cells (Table 1). In this system, a EGFP fluorescence can only be detected when an interaction between the two partner proteins occurs and EGFP reassembles.

**Table 1:** Bacterial plasmids used in this study.

Plasmid	Relevant characteristics	Source or reference
pZM2	pET15b expressing His_6_-NFS1Δ1-55, Amp^R^	[31]
pUMT13	pET15b expressing His_6_–NFS1Δ1-55^C381A^, Amp^R^	This study
pZM4	pACYCDuet1 expressing ISD11, CM^R^	[31]
pZM13	*MOCS3* gene cloned into *Sal*I/*Bam*HI sites of pECFP-C1, generating a ECFP-MOCS3 fusion	This study
pZM143	*MOCS3* gene cloned into *Xho*I/*Bam*HI sites of pEGFP^1–157^, generating a MOCS3-EGFP^1–157^ fusion	This study
pZM144	*MOCS3* gene cloned into *Xho*I/*Bam*HI site of pEGFP^157–238^, generating a MOCS3-EGFP^158–238^ fusion	This study
pZM154	*MOCS3-MoeBD* gene fragment cloned into *Xho*I/*Bam*HI sites of pECFP-N1, generating aMOCS3-MoeBLD-ECFP fusion	This study
pZM101	*MOCS3-RLD* gene fragment cloned into *Xho*I/*Hin*dIII sites of pECFP-C1, generating a ECFP-MOCS3-RLD fusion	This study
pZM29	*NFS1* gene cloned into *Xho*I/*Bam*HI sites of pECFP-N1, generating a NFS1-ECFP fusion	This study
pZM30	*NFS1* gene cloned into *Xho*I/*Bam*HI sites of pEYFP-N1, generating a NFS1-EYFP fusion	This study
pZM145	*NFS1* gene cloned into *Xho*I/*Bam*HI sites of pEGFP^1–157^, generating a NFS1-EGFP^1–157^ fusion	This study
pZM146	*NFS1* gene cloned into *Xho*I/*Bam*HI sites of pEGFP^157–238^, generating a NFS1-EGFP^158–238^ fusion	This study
pZM26	*NFS1*Δ*1-55* gene fragment cloned into *Xho*I/*Bam*HI sites of pECFP-C1, generating a ECFP-NFS1Δ1-55 fusion	This study
pZM27	*NFS1*Δ*1-55* gene fragment cloned into *Xho*I/*Bam*HI sites of pEYFP-C1, generating a EYFP-NFS1Δ1-55 fusion	This study
pZM148	*NFS1*Δ*1-55* gene fragment cloned into *Xho*I/*Bam*HI sites of pEGFP^157–238^, generating aNFS1Δ1-55-EGFP^158–238^ fusion	This study
pZM41	*ISD11* gene cloned into *Xho*I/*Hin*dIII sites of pECFP-N1, generating a ISD11-ECFP fusion	This study
pZM149	*ISD11* gene cloned into *Xho*I/*Hin*dIII sites of pEGFP^1–157^, generating a ISD11-EGFP^1–157^ fusion	This study
pTYB2-MOCS2A	pTYB2 expressing MOCS2A-Intein, Amp^R^	[22]
pGG101	pQE60 expressing MoaD-MoaE, Amp^R^	[62]
pGG130	pTYB2 expressing MoaD-Intein, Amp^R^	[62]
pMMC2	pEYFP-N1 expressing EYFP-MOCS2A fusion in HeLa cells, Kan^R^/Neo^R^	[26]
pMMC30	pFastBac1 expressing MOCS3, Amp^R^	[26]

**Figure 4:**
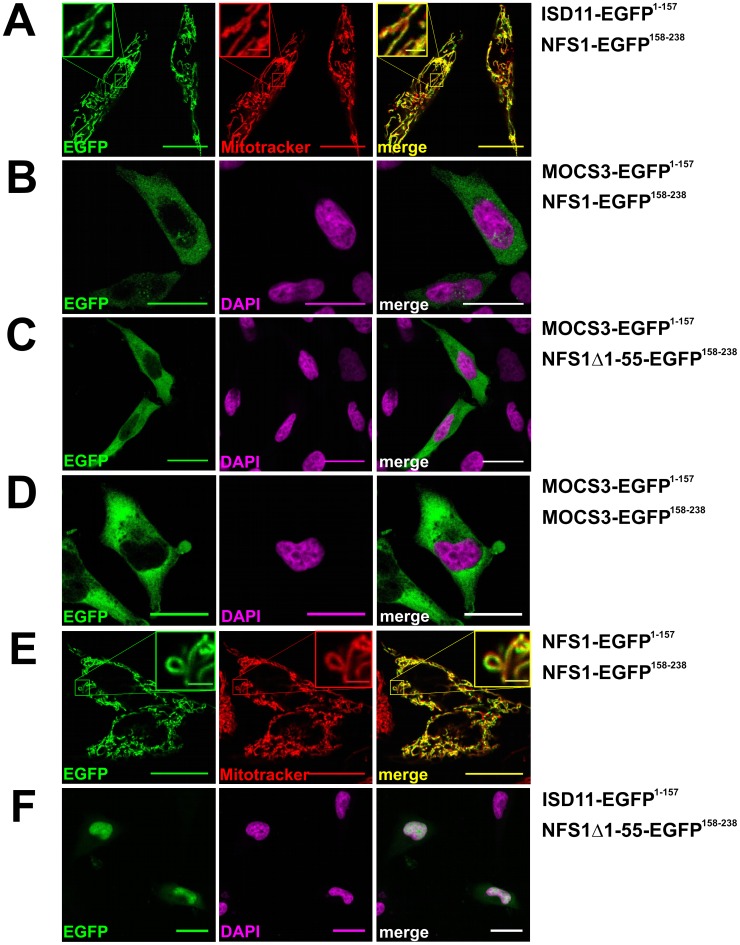
Analysis of NFS1 and MOCS3 interactions by using the split-EGFP system in HeLa cells. Subcellular EGFP assembly of different split-EGFP fusion proteins was analyzed in HeLa cells by confocal fluorescent microscopy. The following fusion proteins were expressed after cotransfection (assembly of EGFP^1–157^ and EGFP^158–238^ resulted in a *green pseudocolor*): *A*, ISD11-EGFP^1–157^ and NFS1-EGFP^158–238^; *B*, MOCS3-EGFP^1–157^ and NFS1-EGFP^158–238^; *C*, MOCS3-EGFP^1–157^ and NFS1Δ1-55-EGFP^158–238^; *D*, MOCS3-EGFP^1–157^ and MOCS3-EGFP^158–238^; *E*, NFS1-EGFP^1–157^ and NFS1-EGFP^158–238^; *F*, ISD11-EGFP^1–157^ and NFS1Δ1-55-EGFP^158–238^. Mitochondria of HeLa cells were visualized with MitoTracker® DeepRed (*red*) or the nuclei were visualized with DAPI stain (*magenta*). Merged pictures are shown right (either resulting in a *yellow* or *white color*). Scale bars, 20 µm; scale bars in the insets, 2 µm.

As shown in Figure 4, using this system we were able to obtain EGFP fluorescence with NFS1-EGFP^158–238^ and ISD11-EGFP^1–157^ in mitochondria or with NFS1-EGFP^158–238^ and MOCS3-EGFP^1–157^ in the cytosol (Figure 4A and B). A higher fluorescence in the cytosol was obtained when MOCS3-EGFP^1–157^ and NFS1Δ1-55-EGFP^158–238^ were cotransfected (Figure 4C). In addition, a EGFP fluorescence was obtained when MOCS3-EGFP^1–157^ and MOCS3-EGFP^158–238^ or NFS1-EGFP^1–157^and NFS1-EGFP^158–238^ were cotransfected, showing that these proteins form homodimers in the cytosol (Figure 4D and E). Using Mitotracker and DAPI staining, we visualized the mitochondria or the nucleus. When ISD11-EGFP^1–157^ and NFS1Δ1-55-EGFP^158–238^ were cotransfected, we mainly detected an EGFP fluorescence in the nucleus. In contrast to our colocalization data shown above, we also observed a weak cytosolic EGFP fluorescence in some cells. This suggests that NFS1Δ1-55 and ISD11 interact in the cytosol when NFS1Δ1-55 is abundantly expressed in the cytosol (Figure 4F). However, since only a few cells showed this fluorescence, it has to be further confirmed in the future whether ISD11 is localized in the cytosol.

In our controls, no specific fluorescence was detected when the EGFP fragments with or without fusion were co- or separately expressed (see supplemental Figure S2). These results show that NFS1 interacts with MOCS3 in the cytosol.

### Detection of Cellular Protein-protein Interactions by Determination of the ECFP Donor Lifetime

Furthermore, we determined the interaction of NFS1Δ1-55 with MOCS3 by analyzing the FRET between ECFP/EYFP fusion proteins in HeLa cells. Like in the fluorescent localization studies shown above the same N-terminal-tagged ECFP/EYFP fusion proteins of NFS1Δ1-55 and MOCS3 were used (see also Table 1). The FRET efficiency of the interactions was calculated by determining the decrease in the ECFP donor lifetime. The lifetime was determined with time-gated FLIM exciting ECFP at 355 nm and recording its fluorescence decay in an overall time interval of 25 ns. The decay curves were fitted bi-exponentially with a long lifetime component of τ_1_ = 3.5±0.3 ns and a short lifetime of τ_2_ = 1.5±0.3 ns (N = 30). The values obtained here using HeLa cells were comparable with the values obtained for ECFP in COS cells or in HeLa cells [34], [35].

**Figure 5:**
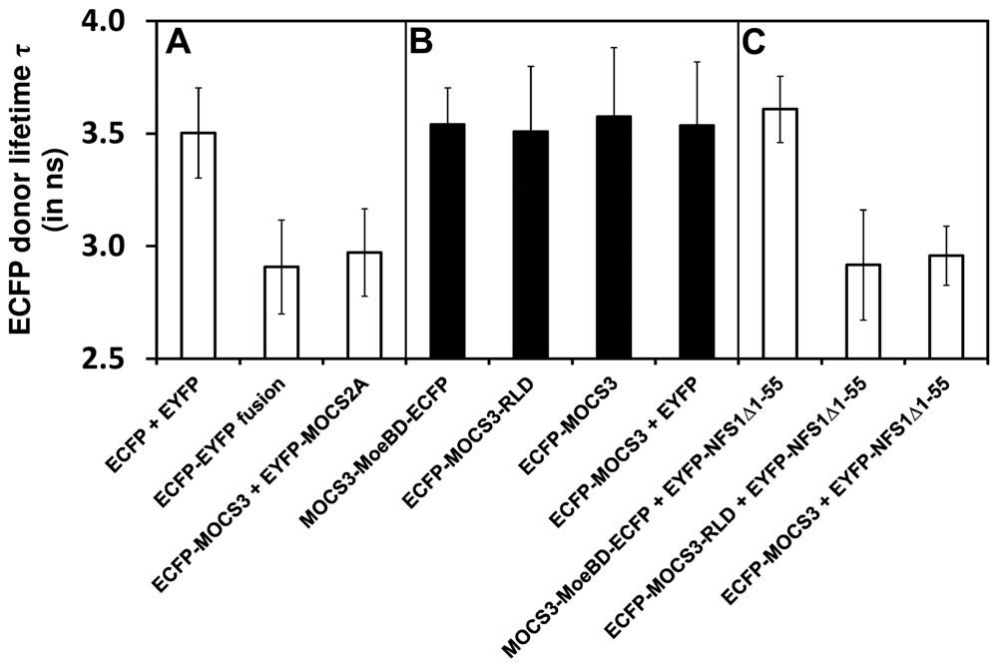
Quantification of the ECFP donor fluorescence lifetime (τ_1_) by time-resolved FRET in HeLa cells. After expression of ECFP and EYFP-tagged proteins in HeLa cells, the ECFP donor lifetime was determined by FRET measurements and the long lifetime component of the bi-exponentially decaying ECFP is shown. *A*, ECFP donor controls, showing expression of ECFP-EYFP fusion proteins, ECFP and EYFP, and ECFP-MOCS3 and EYFP-MOCS2A. *B*, ECFP donor lifetime of MOCS3-MoeBD-ECFP, ECFP-MOCS3-RLD, ECFP-MOCS3 and ECFP-MOCS3/EYFP. *C*, ECFP donor lifetime of MOCS3-MoeBD-ECFP, ECFP-MOCS3-RLD, and ECFP-MOCS3 fusion in presence of EYFP-NFS1Δ1-55 is shown. For each value N = 30–42 cells were measured (displayed as mean ± standard deviation).

In this experiment, the interaction of EYFP-NFS1Δ1-55 with either ECFP-MOCS3 or with the separate domains of MOCS3, MOCS3-MoeBD-ECFP and ECFP-MOCS3-RLD, were analyzed the same way as with ECFP alone. As positive control, a protein fusion of ECFP and EYFP with peptide linker was used, for which the long donor lifetime component was reduced to τ_1_ = 2.9±0.2 ns while the short lifetime component of τ_2_ = 0.9±0.2 ns was also decreased (Figure 5A). This corresponds to a decrease in the long lifetime component of 17%. As shown above, we additionally used ECFP-MOCS3 and EYFP-MOCS2A interaction as control and here a reduction of 15% in the long donor lifetime component was obtained. This is consistent with the data of a previous study [26]. In comparison, the coexpression of ECFP and EYFP did not alter the donor lifetimes (τ_1_ = 3.5±0.2 ns, τ_2_ = 1.5±0.2 ns; Figure 5A).

The resulting ECFP donor lifetimes of the cells coexpressing EYFP-NFS1Δ1-55 and the ECFP-tagged MOCS3 or the separate MOCS3 domains are shown in Figure 5B and C. The protein-fusions MOCS3-MoeB-ECFP, ECFP-MOCS3-RLD, ECFP-MOCS3 were transfected alone or together as ECFP-MOCS3/EYFP and showed an ECFP donor lifetime of about τ_1_ = 3.5±0.3 ns (Figure 5B). The coexpression pairs ECFP-MOCS3-RLD/EYFP-NFS1Δ1-55 and ECFP-MOCS3/EYFP-NFS1Δ1-55 resulted in a decrease of the ECFP donor lifetime of τ_1_ = 2.9±0.2 ns, which is about a 17% reduction and comparable to the ECFP-EYFP fusion construct (Figure 5C). In contrast, the ECFP donor lifetime of the MOCS3-MoeBD-ECFP/EYFP-NFS1Δ1-55 pair did not decrease the ECFP donor lifetime showing that these proteins do not interact (Figure 5C). The results are consistent with the data presented above and confirm the interaction of NFS1 and MOCS3 in the cytosol of HeLa cells. Additionally, these results show that the interaction site is the C-terminal RLD of MOCS3 *in vivo*.

### Analysis of Protein-protein Interactions by SPR Measurements

So far, using the purified proteins an interaction was shown between NFS1Δ1-55 and the separately expressed MOCS3-RLD, since active and stable MOCS3 was not present at this time [31]. In a recent study we presented the successful purification of active MOCS3 from Sf9 cells [26]. To analyze the dissociation constant of NFS1Δ1-55 and MOCS3, SPR measurements were employed for real-time detection of specific interactions using the purified proteins.

MOCS3 and MOCS3-RLD were immobilized on a CM5 chip via amine coupling. As binding partners we used purified NFS1Δ1-55, NFS1Δ1-55/ISD11 and NFS1Δ1-55^C381A^/ISD11.

The variant NFS1Δ1-55^C381A^/ISD11 was shown to be inactive. The expression of the NFS1Δ1-55^C381A^ variant without ISD11 led to precipitation of the protein, while the wildtype without ISD11 was stable for two days at 4°C (data not shown).

The mean K_D_ values obtained from three independent SPR measurements for the protein pairs are listed in Table 2, while the SPR binding curves are shown in the Figure S3 in the supplemental information. The data show that NFS1Δ1-55 interacted with immobilized MOCS3 with a K_D_ value of 28.3±3.1 nM. When ISD11 was in complex with NFS1Δ1-55 the binding affinity decreased for MOCS3 showing a K_D_ value of 119±4.7 nM. Thus, ISD11 and MOCS3 might have overlapping binding sites on NFS1. Additionally, K_D_ values in the same range were obtained for MOCS3-RLD showing that NFS1 likely interacts with the C-terminal rhodanese domain of MOCS3.

**Table 2:** Analysis of protein-protein interactions between MOCS3 and NFS1Δ1-55 by SPR measurements.

Immobilized protein^a^	Protein partner b	K_D_ (nM)^c^
MOCS3	NFS1Δ1-55	28.3±3.1
	NFS1Δ1-55/ISD11	119.3±4.7
	NFS1Δ1-55^C381A^/ISD11	218.0±80.7
	*E. coli* IscS	−d
MOCS3-RLD	NFS1Δ1-55	36±7.1
	NFS1Δ1-55/ISD11	166.0±22.5
	NFS1Δ1-55^C381A^/ISD11	241.0±35.5
	*E. coli* IscS	−
BSA	NFS1Δ1-55	−
	NFS1Δ1-55/ISD11	−
	NFS1Δ1-55^C381A^/ISD11	−
	*E. coli* IscS	−

a Proteins were immobilized via amine coupling.

b Proteins were injected using KINJECT protocol, injecting samples in a concentration range of 0.3–10 µM. Flow cells were regenerated by injection of 20 mM HCl.

c K_D_ mean values with standard deviation were obtained from 3 independent measurements after global fitting procedures for 1∶1 binding for each measurement.

d −, binding not calculated.

The mean K_D_ value of the NFS1 variant NFS1Δ1-55^C381A^/ISD11 with MOCS3 was at 218.0±80.7 nM and with MOCS3-RLD at 241.0±35.5 nM. In comparison to NFS1Δ1-55/ISD11, the mean K_D_ values were only slightly decreased. As negative control we used BSA and *E. coli* IscS, which showed either no interaction with MOCS3 or in the case of *E. coli* IscS binding curves that could not be evaluated with the 1∶1 binding model. These results using MOCS3 purified after expression in Sf9 cells confirmed our previously published data using MOCS3-RLD and yeast Uba4 [31], confirming the interaction of NFS1 with the C-terminal domain of MOCS3.

### Reconstitution of the Activity of the Molybdoenzyme Nitrate Reductase in *N. crassa nit-1* Extracts

**Figure 6:**
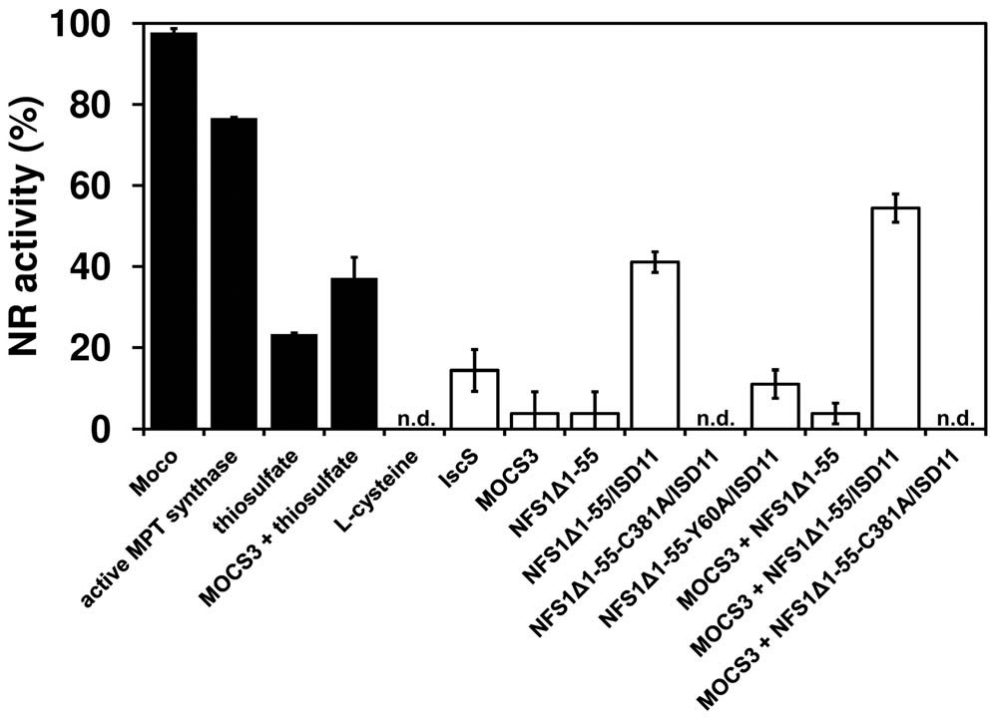
Reconstitution of nitrate reductase activity in *N. crassa nit-1* extracts using different sulfur donors. All reconstitution mixtures contained 30 µl freshly prepared *N. crassa nit-1* extracts, 10 µl 0.5 M sodium molybdate, and 10 µM of MOCS3 and/or NFS1Δ1-55 or an excess of isolated Moco and 10 µM active *E. coli* MPT synthase (positive control). As sulfur source either 1 mM L-cysteine or 1 mM sodium thiosulfate was added. The reaction mixtures were incubated for 30 min at room temperature, then another 20 min for the reaction of the reconstituted nitrate reductase, and finally the reaction was stopped and the produced nitrite was determined at 540 nm. *n.d*., not detectable.

To further support the observation that NFS1 acts as the sulfur donor for MOCS3, we analyzed the reconstitution of assimilatory NADPH nitrate reductase (NR) using the *N. crassa nit-1* extracts [36]. This reconstitution assay can be used to detect the presence of MPT or Moco in samples [37], [38]. The assay uses freshly prepared extracts of the *N. crassa nit-1* mutant as a source of apo-NR which lacks Moco and is therefore inactive. The *nit-1* mutant is known to accumulate cPMP which is the first stable intermediate of Moco biosynthesis [39]. The position of the mutation of *nit-1* has not been mapped to date; however, since the addition of MPT, Moco, sulfurated MPT synthase or sulfide as sulfur source restores NR activity, it shows that the sulfur donor for MPT synthesis is likely affected in the *nit-1* strain [36], [37], [40]. We set the reconstitution of *nit-1* NR activity with isolated Moco to 100% (Figure 6). MOCS3 showed no ability to restore NR activity alone (data not shown), but a reconstitution of 37% NR activity was obtained with thiosulfate as sulfur source (Figure 6). The NFS1Δ1-55/ISD11 complex was able to reconstitute the apo-NR activity up to 41% in the presence of L-cysteine. About 54% restored NR activity was obtained when MOCS3, NFS1Δ1-55/ISD11 and L-cysteine were added to the *nit-1* extract, showing that NFS1 is able to transfer the sulfur to MOCS3. The NFS1 variant NFS1Δ1-55^C381A^/ISD11 was inactive and was not able to restore the NR activity. L-cysteine and thiosulfate alone did not show any reconstitution. These data clearly show that NFS1 is able to reconstitute the activity of the *N. crassa nit-1* NR, and the reconstitution of NR was further increased when MOCS3 was added.

## Discussion

In this report we have shown that human NFS1 is the sulfur donor for MOCS3 in the cytosol. Fractionation of HeLa cells and immunodetection were used to detect the localization of NFS1 in the cytosol (Figure 1). By this method, the additional localization of NFS1 in mitochondria and the nucleus was confirmed [4], [5], [19], [20], [32], [33].

For yeast Nfs1 a two-step processing in the mitochondrial matrix, first by the mitochondrial processing peptidase and then by the peptidase Icp55 was suggested [20]. Whether yeast Icp55 processes Nfs1 outside of the mitochondria is unknown, but it was clearly shown that both proteins are targeted to the nucleus and that the size of mature yeast Nfs1 is not changed among the two compartments [20]. However, the role of Nfs1 in the nucleus remains unclear so far. Additionally, it is not known whether a similar processing mechanism exists for human NFS1. The mechanism how NFS1 remains in the cytosol or is targeted back from mitochondria is not resolved so far. Only a few mechanisms for the eclipsed distribution of dual localized proteins in the cell have been suggested previously [41], [42], [43].

In more detailed studies by using a split-EGFP system and FRET studies, we showed that NFS1 interacts with MOCS3 in the cytosol (Figure 4, 5, S2 and S4). In FLIM-FRET studies the donor fluorescence lifetime of ECFP was determined as an indication for the strength of the interaction of the protein pairs. In these studies, MOCS3 and NFS1Δ1-55 were coexpressed as ECFP/EYFP fusion proteins in HeLa cells to determine the FRET between the fluorescent dyes. Because of the high dependence of the FRET signal on the distance between the dyes, only the complex formation between NFS1 and the candidates enables an energy transfer. Thus, by the interaction of the proteins we suggest that NFS1 is the sulfur donor for MOCS3 in the cytosol.

In all eukaryotes, MOCS3 homologues contain an N-terminal MoeB/E1-like domain with the C-terminal rhodanese-like domain. Recently it was shown that MOCS3 has a dual role in the cell by transferring the sulfur from the C-terminal RLD to two acceptor proteins MOCS2A and URM1 [26] (see also Figure S4). MOCS2A is involved in Moco biosynthesis and interacts with MOCS2B. MOCS2B binds the Moco intermediate cPMP and two sulfur atoms are sequentially added from the C-terminal thiocarboxylate group of two MOCS2A molecules, thus forming MPT (Figure S4). URM1, in contrast, is involved in the thiolation tRNAs for mcm^5^s^2^U_34_ modification on tRNAs for Lys, Gln and Glu at their wobble positions (Figure S4). In contrast, in yeast the MOCS3 homologue Uba4 has only one partner, Urm1, since *S. cerevisiae* does not contain molybdoenzymes or the genes for Moco biosynthesis like MOCS2A [44]. Thus, the yeast MOCS3-homologue Uba4 only interacts with Urm1 in the cytosol for 2-thiouridine formation on tRNAs [25]. In yeast, however, Nfs1 has not been identified in the cytosol so far. In a reverse genetic approach combined with mass spectrometry, the yeast Tum1 protein was identified to be involved in tRNA thiolation [25]. Tum1 is a tandem rhodanese-like protein which was shown to be localized in the cytosol and mitochondria. Thus, for yeast it has been suggested that Tum1 interacts with Nfs1 in mitochondria, and afterwards sulfurated Tum1 is exported to the cytosol where it can interact with Uba4 for sulfurtransfer. It was suggested that Tum1 is a shuttling protein between mitochondria and the cytosol [23], [25]. This hypothesis is supported by the observation that following depletion of Nfs1 in yeast, the thiomodification of cytosolic tRNAs was shown to be somehow delayed, suggesting that a required component is transported from the mitochondria to the cytosol [17].

TUM1 has not been characterized in humans so far, thus, an involvement in tRNA thiolation remains unclear. However, in humans the situation is different, since MOCS3 is required for both tRNA thiolation and Moco biosynthesis in the cytosol. Moco was shown to be essential for humans, being the catalytic center of sulfite oxidase [45] (Figure S4). The absence of sulfite oxidase results in severe neurological defects which usually leads to death in early childhood [46]. A delay in sulfurtransfer from the mitochondria to the cytosol might be not beneficial for Moco biosynthesis in humans, thus, NFS1 resides in low amounts in the cytosol for direct interaction with MOCS3 thereby accelerating sulfurtransfer.

However, it has also been suggested that in the mitochondria an iron and/or sulfur-containing component is synthesized that is exported to the cytosol by the help of an ABC transporter of the mitochondrial inner membrane [6]. This transporter in addition to the intermembrane space protein Erv1 and gluthatione are required for cytosolic FeS cluster biosynthesis [6], [47], [48]. These studies are based on the finding that the mitochondrial FeS cluster machinery is required for the cytosolic FeS cluster assembly (CIA) pathway. The research field on the CIA pathway is still emerging. Basically, the initiation of the cytosolic FeS cluster assembly requires the apo-Cfd1-Nbp35 scaffold complex which depends on the iron and sulfur containing component from the mitochondria in addition to the factor Dre2 [49], [50], [51], [52]. Nar and Cia1 were shown to interact with the Cfd1-Nbp35 complex, facilitating cluster transfer to cytosolic and nuclear FeS proteins [53], [54].

However, the components of the mitochondrial ISC machinery ISCU, NFU1, HSC20, and FXN were also detected in the cytosol [5], [21], [32], [55], [56], [57]. Thus, in addition to NFS1, which was identified in the cytosol in this study, the synthesis of FeS clusters by these proteins would be possible in the cytosol. However, a function for the cytosolic human NFS1 in *de novo* assembly of cytosolic FeS proteins could not be shown in the absence of mitochondrial NFS1 [33]. Likewise, cytosolic human ISCU is not sufficient to *de novo* assemble cytosolic/nucleic FeS proteins, but it has been suggested that the protein might play a role in FeS cluster repair after oxidative damage or iron deprivation [58]. Also, cytosolic isoforms of frataxin have been found and described to restore cytosolic aconitase and IRE-binding activity of IRP1 to normal levels in cells derived from Friedreichs ataxia patients or after causing oxidative stress, while the mitochondrial aconitase activity was unaltered [57], [59], [60]. Conclusively, the role of ISCU, NFU1, HSC20, and FXN in the cytosol still remains to be further elucidated.

In this report, the proposed involvement of NFS1 in conjunction with MOCS3 in Moco biosynthesis is supported by studies making use of the *N. crassa nit-1* extract (Figure 6) [36], [37]. This mutant lacks the activity of the molybdoenzyme nitrate reductase (NR) due to an impairment in the sulfurtransfer reaction to MPT synthase, which converts cPMP to MPT. The *nit-1* strain thus accumulates cPMP [39], [61]. Using *nit-1* extracts, NR activity can be restored by the addition of either Moco, active MPT synthase or a direct sulfur donor like sulfide. In our assay, the addition of NFS1Δ1-55/ISD11 together with L-cysteine reconstituted the activity of apo-NR. This shows that NFS1 was able to act as a sulfur donor for MPT synthase. The reconstitution of NR activity was further increased in the presence of MOCS3. This showed that also in *N. crassa*, NFS1 was able to provide sulfur for the MPT synthase reaction directly to MOCS3 with the highest activity. A common mechanism in sulfurtransfer for Moco biosynthesis in eukaryotic systems is likely (Figure S4), with yeast being an exception due to the lack of Moco biosynthesis.

While our results show that NFS1 is located in the cytosol where it is used for Moco biosynthesis, the involvement of ISD11 in this reaction still remains unclear. So far, ISD11 was described as a stabilization factor of NFS1 which is essential for its activity in FeS cluster formation in mitochondria (see Figure S4). NFS1 is prone to aggregation and FeS clusters cannot be formed when functional ISD11 is absent in the cell [7], [8].

Our localization studies using ECFP and EYFP fusions localized ISD11 mainly in mitochondria and the nucleus (Figure 2, 3, S1 and S4). Previous studies using ISD11 antibody did not detect an additional localization in the cytosol [21]. We were also unable to detect an interaction of ISD11 with NFS1 in the cytosol by using the split-EGFP system (Figure 4). Only when NFS1Δ1-55 is coexpressed with ISD11 a weak EGFP fluorescence was observed in the cytosol of some cells (Figure 4). However, for future studies it would be interesting to investigate the role for NFS1 and ISD11 in the nucleus as well as to clarify whether ISD11 is involved in Moco biosynthesis in the cytosol.

## Materials and Methods

### Protein Expression and Purification

Human NFS1Δ1-55 and NFS1Δ1-55^C381A^ was expressed in the presence or absence of human ISD11 in *E. coli* BL21(DE3) cells from the plasmids pZM2, pUMT13 and pZM4, and purified as described previously [31]. Human MOCS2A was expressed in *E. coli* BL21(DE3) using the plasmid pTYB2-MOCS2A and purified as previously described by Leimkühler *et al*. [28]. Active *E. coli* MPT synthase (composed of MoaE and MoaD) was expressed in *E. coli* from the plasmids pGG110 and pGG130 and purified as described previously by Gutzke *et al.* [62]. Human MOCS3 was expressed in Sf9 cells and purified as previously described by Chowdhury *et al*. [26].

### Cell Culture Maintenance

HeLa cells were cultured in Dulbecco’s modified Eagle’s medium (DMEM, PAN-Biotech, Germany) supplemented with 10% fetal bovine serum (FBS, PAN-Biotech, Germany) and glutamine. Cell cultures were maintained at a temperature of 37°C and 5% CO_2_ atmosphere. For localization and FRET analyses, HeLa cells were grown on poly-L-lysine coated coverslips prior to transfection.

### Subcellular Fractionation and Immunoblotting

For fractionation of soluble and membrane-associated proteins, HeLa cells were grown in at least six 75 cm^2^-sized cell culture flasks until 80–90% confluence (at least 50.4×10^6^ cells), harvested by trypsination, centrifuged and washed once with pre-warmed PBS. Freshly harvested HeLa cells were resuspended and incubated for 8 min at 4°C in a volume of ice-cold cell lysis buffer (10 mM Tris/HCl, pH 7.4, 1.5 mM MgCl_2_, 10 mM KCl, 250 mM sucrose, 1 mM DTT, 1 mM EDTA, 1 mM EGTA, 0.007% digitonin, and protease inhibitor cocktail) equal to 30 times the volume of the pellet. Lysates were centrifuged at 1,000×g and 4°C for 5 min. The supernatant of the lysate corresponding to soluble cytosolic proteins was subjected to another centrifugation step at 100,000×g for 1 h and 4°C to remove residual membranes and was designated as the fraction cytosol. The cytosolic fraction was concentrated at least 32-fold by ultrafiltration, using a molecular weight cut-off of 10 kDa. For isolation of mitochondria, an optimized protocol for HeLa cells was used following the instructions of Wieckowski *et al.* [63]. To obtain the nuclear fraction we followed the instructions of Hinz *et al.* [64]. All fractions were frozen in liquid nitrogen and stored at −80°C.

For immunoblot analysis, proteins of the fractions were separated on 12% SDS-PAGE and transferred to PVDF membranes. Proteins were detected by immunoblotting with the indicated primary antibodies (1∶1000 anti-NFS1, 1∶1000 anti-MOCS3, 1∶5000 anti-γ-actin, 1∶1000 anti-ABCB7 and 1∶1000 anti-citrate synthase were obtained from Sigma; 1∶2000 anti-laminB1 was obtained from Abcam) and peroxidase-conjugated secondary antibodies (1∶1000 anti-rabbit: Thermo; 1∶5000 anti-mouse: Sigma). Blots were developed with an enhanced chemiluminescence (ECL) detection system (Super Signal; Thermo, Pierce). The protein content in the samples was estimated by using the Bradford reagent with bovine serum albumin (BSA) as a standard.

### Subcellular Interaction Studies Using the Split-EGFP System

Coding regions for NFS1, NFS1Δ1-55, ISD11, and MOCS3 were amplified by PCR by using pZM2, pZM4 [31] and pZM13 [26] as template and the obtained fragments were cloned into the mammalian expression vectors pEGFP^1–157^-N1 and pEGFP^158–238^-N1 [65], resulting in the plasmids pZM143, pZM44 (MOCS3), pZM145, pZM146 (NFS1), pZM148 (NFS1Δ1-55), and pZM149 (ISD11), respectively (Table 1).

Proteins were C-terminally fused to either EGFP^1–157^ or EGFP^158–238^ and expressed in HeLa cells. For the expression, HeLa cells were transiently transfected with corresponding plasmids (see Table 1) using Lipofectamin® (Invitrogen). For staining of mitochondria and nuclei Mitotracker DeepRed® (Invitrogen) (1∶10,000) and DAPI (Sigma) (1∶1000) was used. 12 h after transfection, cells were fixed for 40 min using 4% paraformaldehyde in PBS. The cells were washed twice with PBS and mounted onto slides with Mowiol (Roth). Images for EGFP fluorescence was imaged with a confocal microscope LSM710 (Carl Zeiss Microscopy, Jena, Germany) equipped with a EC Plan-Neofluar 40× Oil objective having a numerical aperture (NA) of 1.3. The EGFP, DAPI and Mitotracker were excited sequentially (multi-track mode) at 488 nm, 405 nm, and 633 nm, respectively. Images were taken with a depth of 12 bit in the spectral range of the emission at 493–612 nm (for EGFP), 413–560 nm (for DAPI) and 637–735 nm (for Mitotracker), respectively. The imaging software ZEN2009 was used for operating the system and image acquisition. For processing the ImageJ (MacBiophotonics) program was used.

### Subcellular Localization and FRET Analysis

For Förster resonance energy transfer (FRET) analyses and cellular localization studies in human HeLa cells fusion proteins with the enhanced yellow fluorescent protein (EYFP) or the enhanced cyan fluorescent protein (ECFP) were used. Coding regions of NFS1, NFS1Δ1-55, ISD11, MOCS3, MOCS3-RLD, and MOCS3-MoeB-like domain were amplified by PCR using pZM2, pZM4 [31], and pZM13 as template and cloned into the mammalian expression vectors pEYFP-N1, pECFP-N1, pEYFP-C1 or pECFP-C1 (Clontech) for FRET and subcellular localization studies, resulting in the corresponding plasmids pZM27 (NFS1Δ1-55), pZM30 (NFS1), pZM41 (ISD11), pZM101 (MOCS3-RLD), and pZM154 (MOCS3-MoeBD), respectively (Table 1). The plasmid pMMC2, containing MOCS2A in pEYFP-C1 was described previously [26]. For the localization studies HeLa cells were stained and fixed as described above. Images were taken with a confocal microscope LSM710 by using a PlanApo 1.4/63× Oil or EC Plan-Neofluar 1.3/40× Oil objective.

EYFP and ECFP were excited sequentially (multi-track mode) at 514 nm and 458 nm. Images were taken with a depth of 12 bit in the spectral range of the emission at 519–578 nm (for EYFP) and 462–510 nm (for ECFP). The imaging software ZEN2009 was used for operating the system and image acquisition. For processing the ImageJ (MacBiophotonics) program was used.

For FRET analysis HeLa cells were transiently transfected using a modified calcium phosphate method described previously in [26]. FRET requires an overlap of the emission spectrum of the donor, here ECFP, and the absorption spectrum of the acceptor, here EYFP. In addition, the FRET pair has to be in close spatial proximity and appropriate orientation. The donor fluorescence of ECFP is comparably dim, and sensitized emission of the acceptor is hard to detect due to the spectral bleed through of ECFP fluorescence. Thus, the fluorescence lifetime of the donor was chosen as parameter to monitor FRET performing fluorescence lifetime imagine microscopy (FLIM).

For FLIM, a Visitron Systems imaging system based on an inverted microscope (Axio Observer Z1, Carl Zeiss Microscopy) equipped with a Plan-NeoFluar 0.75/40× objective was used. ECFP was excited with a mode-locked ps-pulsed Nd-YAG laser with regenerative amplifier (PL2201/TH, Ekspla). The 3^rd^ harmonic output at 355 nm and a repetition rate of 1 kHz was 30 µJ/pulse. The output of the Nd-YAG laser was coupled free space without collimation optics into a 500 µm quartz fibre bundle which was then coupled into the microscope. Inside the microscope, a 355 nm dichroic mirror and a 400 nm long-pass filter were used for separating excitation and fluorescence emission light. For spectral separation of ECFP and EYFP emissions, a Dual View emission splitter (Photometrics) was placed in front of the detector, allowing analysis of two emission channels (485/30 nm, 540/30 nm). For time-gated detection an iCCD camera (Pimax2, Princeton Instruments) was used. For all measurements, 100 frames with a time increment of 0.25 ns and 1.8 ns gate width were acquired to monitor an overall time interval of 25 ns. For each single frame, 250 pulses were integrated on a chip. In order to synchronize the iCCD camera to the laser, the Q-switch monitor output of the laser was used to trigger the TTL input of the CCD control unit.

Corresponding reference images were acquired with a multi point confocal scanner (Infinity 3, VisiTech), which was also connected to the imagine system. Samples were excited with an Ar-Kr ion laser (Innova 70C, Coherent) at 456 nm for ECFP or 514 nm for EYFP and images were recorded by a CoolSnap HQ^2^ CCD camera (Photometrics).

Data acquisition and processing were performed with WinView 2.5 (Roper Scientific) and Metamorph 7.2 (Molecular Devices). For each sample N = 30–42 cells were measured. The efficiency of FRET was determined by measuring the fluorescence donor lifetime of ECFP alone (τ_D_), and in the presence of an acceptor (τ_DA_). The decay curves were fitted bi-exponentially with a long lifetime component τ_1_ and a short lifetime of τ_2_ of both τ_D_ and τ_DA_ (in ns).

### Surface Plasmon Resonance (SPR) Measurements

All binding experiments were conducted on a SPR based Biacore^TM^T200 instrument on CM5 senor chips at a temperature of 25°C and a flow of 30 µL/min using the evaluation T200 software (GE, Uppsala, Sweden). The autosampler rack containing the samples was cooled throughout the entire measurements to 8°C. The response units (RU) for the immobilization of the proteins per flow cell of three independent experiments were between 400–600. As running buffer, 20 mM phosphate, 150 mM NaCl, 0.005% (v/v) Tween 20, pH 7.4 was used. NFS1Δ1-55 and NFS1Δ1-55/ISD11 variants with concentrations of 0.31, 0.63, 1.25, 2.5, 5 and 10 µM were injected for 4.5 min at a flow rate of 30 µL/min followed by a 15 min dissociation using the kinject command and regeneration of the senor surface with 20 mM HCl for 1 min. As controls BSA was additionally immobilized and *E. coli* IscS and BSA were used as additional analyte. Binding curves were corrected by substraction of buffer injection curves for both flow cells.

### Apo-nitrate Reductase Reconstitution with NFS1

*Neurospora crassa nit-1* mutant is known to accumulate cPMP and lacks the activity of molybdodenzymes like nitrate reductase. *N. crassa* was grown as described previously [36], [66]. For production of *nit-1* extracts, 2 ml of ice-cold 100 mM K-phosphate, pH 7.4, containing 1 mM DTT, 5 mM EDTA, 1 mM PMSF were added per 1 g wet weight of mycelia and grinded for 5 min at 4°C using mortar and pestle. The crude extract was centrifuged at 4°C and 17,000×g for 20 min to remove cell debris. All following reactions were performed at 25°C. The apo-nitrate reductase (apo-NR) reconstitution was carried out in a total volume of 100 µl containing 30 µl *nit-1* extract, 50 mM sodium molybdate, and 10 µM of each protein (MOCS3, *E. coli* IscS, human NFS1 variants, or *E. coli* MPT synthase). As additional sulfur source 1 mM sodium thiosulfate or L-cysteine was used. To test for the quality of the produced *nit-1* extract Moco was added to the extracts. Moco was freshly isolated from human sulfite oxidase under anaerobic conditions following the instruction in [67]. After 30 min of reconstitution, the holo-NR activity was tested by the addition of 350 µl of 0.4 M KH_2_PO_4_, 0.2 M NaNO_3_, 0.1 mM FAD, 50 mM Na_2_SO_3_ and 50 µl 2 mM NADPH. The reaction was stopped after 20 min by the addition of 500 µl 1% sulfanilamide (in 25% HCl). For detection of formed nitrite, 500 µl 0.09% N-(1-naphthyl)ethylenediamine hydrochlorid was added and incubated for another 20 min. The developed color enables the detection by the absorbance at 540 nm. Therefore, the samples were centrifuged for 5 min at 12,000×g and the nitrite was quantified in the protein-free supernatants.

## Supporting Information

Figure S1 **Detection of mitochondrial targeting of NFS1 and ISD11 in HeLa cells.** The cells shown here are identical with the cells shown in Figure 2A. Subcellular colocalization of (*A*) NFS1-EYFP and (*B*) ISD11-ECFP fusion proteins with the mitochondrial pattern was analyzed in HeLa cells by fluorescent confocal microscopy. The fluorescence of the fusion proteins are presented as *green pseudocolor*, while the stain of the mitochondria with Mitotracker is shown in *red pseudocolor*. Colocalization between fusion proteins and mitochondrial pattern is shown *right* which resulted in a *yellow color*; Scale bars, 20 µm.
(TIF)

Figure S2 **Controls for the split-EGFP interaction analysis.** The following fusion proteins were expressed after transfection (assembly of EGFP^1–157^ and EGFP^158–238^ resulted in a *green pseudocolor*): *A*, MOCS3-EGFP^1–157^; *B*, NFS1-EGFP^158–238^; *C*, NFS1Δ1-55-EGFP^158–238^; *D*, ISD11-EGFP^1–157^; *E*, MOCS3-EGFP^1–157^ and EGFP^158–238^; *F*, EGFP^1–157^ and NFS1Δ1-55-EGFP^158–238^. Mitochondria of HeLa cells were stained with MitoTracker® DeepRed (second row, *red pseudocolor*) and the nuclei were stained with DAPI stain (third row, *blue pseudocolor*). Merged pictures are shown right (the merge of *green* and *red pseudocolor* would result in a *yellow color*). Scale bars, 20 µm.
(TIF)

Figure S3 **Analysis of the interaction between NFS1 and MOCS3 by SPR.** Shown are Biacore sensograms of interactions between the immobilized proteins MOCS3 (left row) and MOCS3-RLD (right row) and varying concentrations of the ligands *A*, NFS1Δ1-55; *B*, NFS1Δ1-55/ISD11; *C*, NFS1Δ1-55^C381A^/ISD11; D, *E. coli* IscS; and *E*, BSA. Binding curves were corrected by substraction of buffer injection curves for both flow cells.
(TIF)

Figure S4 **Model for Moco biosynthesis and FeS cluster biosynthesis in the cell.** NFS1 and ISD11 are predominately targeted to the mitochondria but additionally were detected in the nucleus. In the mitochondria, the NFS1/ISD11 complex is the sulfur donor for FeS cluster biogenesis and for the thiomodification of mitochondrial tRNAs. In the cytosol, NFS1 interacts with the rhodanese-like domain (RLD) of MOCS3 transferring its protein-bound persulfide-sulfur from NFS1-Cys^381^ to MOCS3-RLD-Cys^412^. MOCS3 adenylates MOCS2A and URM1 by its N-terminal MoeB/E1-like domain and further forms a thiocarboxylate on both proteins by sulfur transfer from the C-terminal RLD. URM1 is involved in the thiolation of the wobble base thiouridine to 5-methoxycarbonylmethyl-2-thiouridine (mcm^5^s^2^U^34^) in cytoplasmic tRNAs while MOCS2A forms with MOCS2B the active MPT synthase and transfers the sulfur for the formation of MPT in Moco biosynthesis. Moco is important for the activity of the molybdoenzymes sulfite oxidase, mARC, xanthine dehydrogenase and aldehyde oxidase. The function of NFS1 and ISD11 in the nucleus remains unknown. It is also not clear whether ISD11 has a role in the cytosol.
(TIF)
