# Supplementary figures and images for: The L-Cysteine Desulfurase NFS1 Is Localized in the Cytosol where it Provides the Sulfur for Molybdenum Cofactor Biosynthesis in Humans
Source: PLoS One. 2013 Apr 12;8(4):e60869. doi: 10.1371/journal.pone.0060869 (PMC3625234; doi:10.1371/journal.pone.0060869)

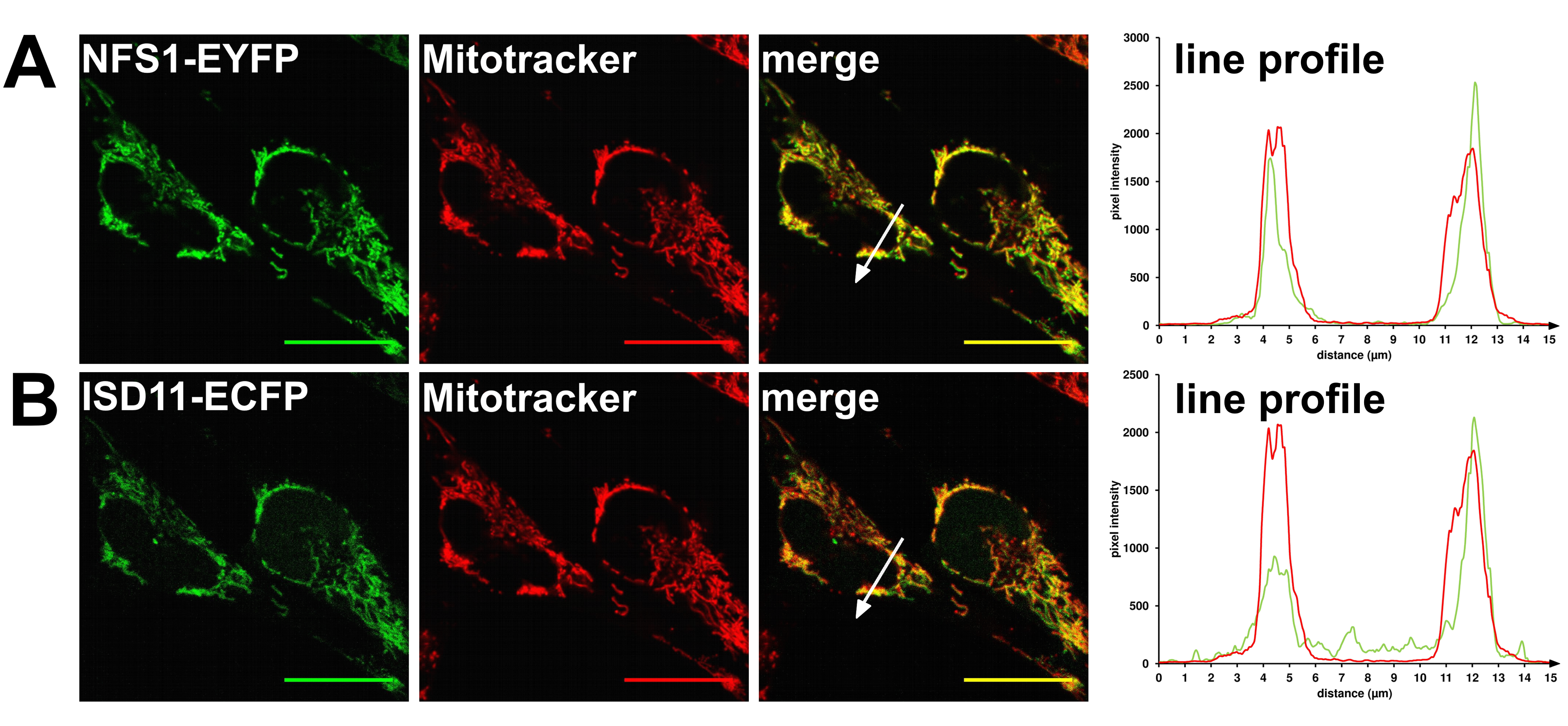

Supplement: Figure S1 — Detection of mitochondrial targeting of NFS1 and ISD11 in HeLa cells. The cells shown here are identical with the cells shown in Figure 2A. Subcellular colocalization of (A) NFS1-EYFP and (B) ISD11-ECFP fusion proteins with the mitochondrial pattern was analyzed in HeLa cells by fluorescent confocal microscopy. The fluorescence of the fusion proteins are presented as green pseudocolor, while the stain of the mitochondria with Mitotracker is shown in red pseudocolor. Colocalization between fusion proteins and mitochondrial pattern is shown right which resulted in a yellow color; Scale bars, 20 µm. (TIF) [file pone.0060869.s001.tif]

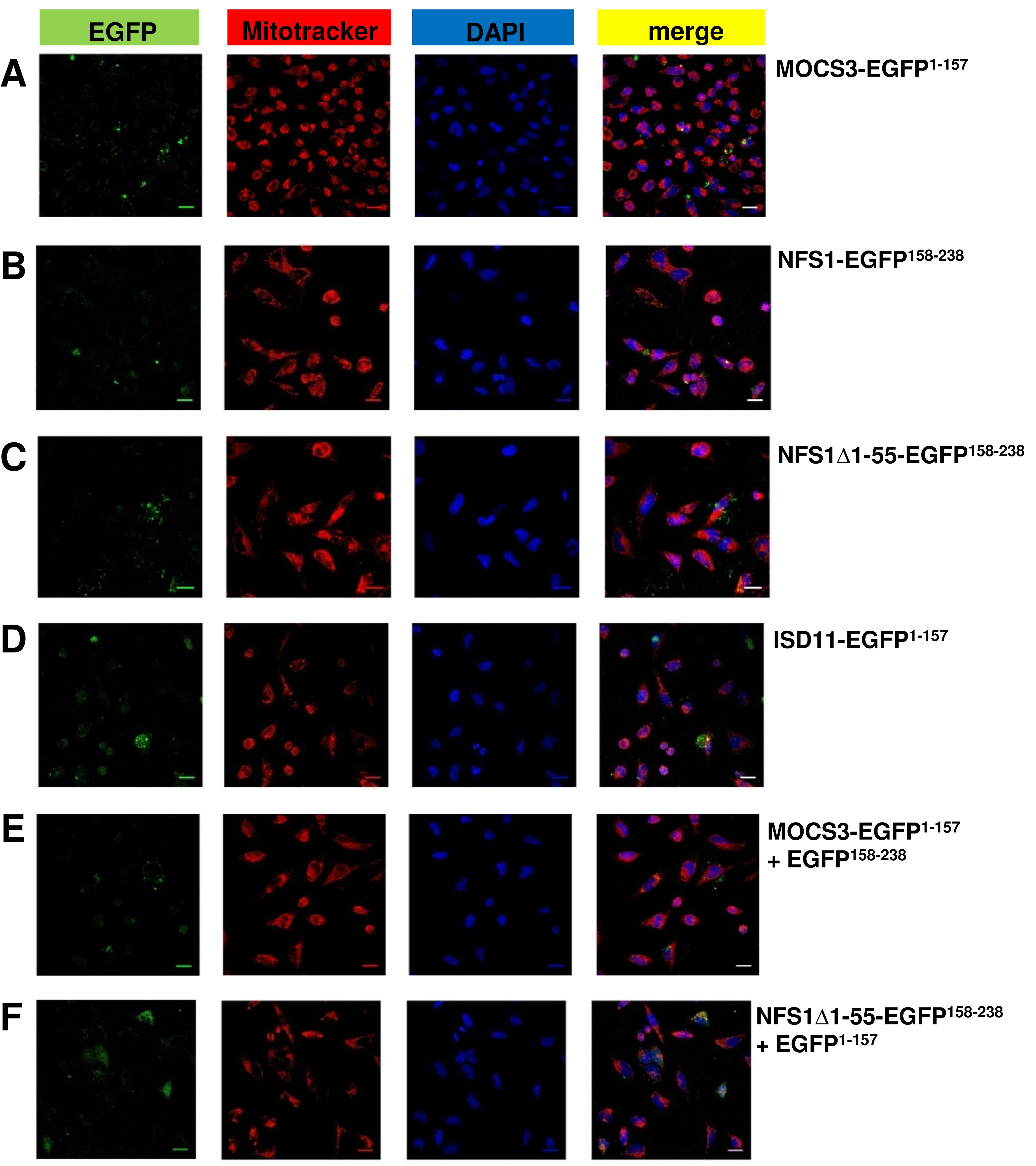

Supplement: Figure S2 — Controls for the split-EGFP interaction analysis. The following fusion proteins were expressed after transfection (assembly of EGFP1–157 and EGFP158–238 resulted in a green pseudocolor): A, MOCS3-EGFP1–157; B, NFS1-EGFP158–238; C, NFS1Δ1-55-EGFP158–238; D, ISD11-EGFP1–157; E, MOCS3-EGFP1–157 and EGFP158–238; F, EGFP1–157 and NFS1Δ1-55-EGFP158–238. Mitochondria of HeLa cells were stained with MitoTracker® DeepRed (second row, red pseudocolor) and the nuclei were stained with DAPI stain (third row, blue pseudocolor). Merged pictures are shown right (the merge of green and red pseudocolor would result in a yellow color). Scale bars, 20 µm. (TIF) [file pone.0060869.s002.tif]

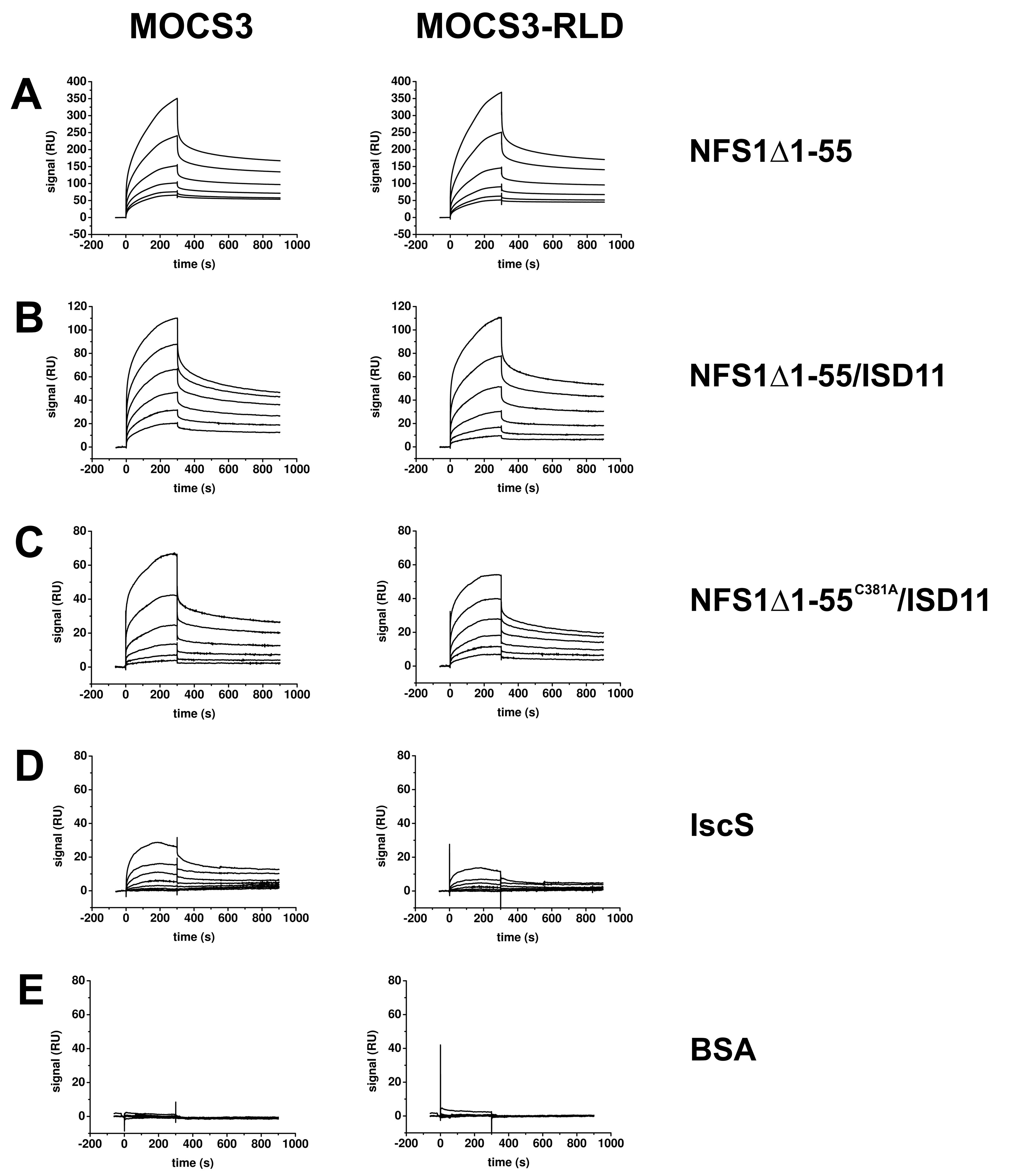

Supplement: Figure S3 — Analysis of the interaction between NFS1 and MOCS3 by SPR. Shown are Biacore sensograms of interactions between the immobilized proteins MOCS3 (left row) and MOCS3-RLD (right row) and varying concentrations of the ligands A, NFS1Δ1-55; B, NFS1Δ1-55/ISD11; C, NFS1Δ1-55C381A/ISD11; D, E. coli IscS; and E, BSA. Binding curves were corrected by substraction of buffer injection curves for both flow cells. (TIF) [file pone.0060869.s003.tif]

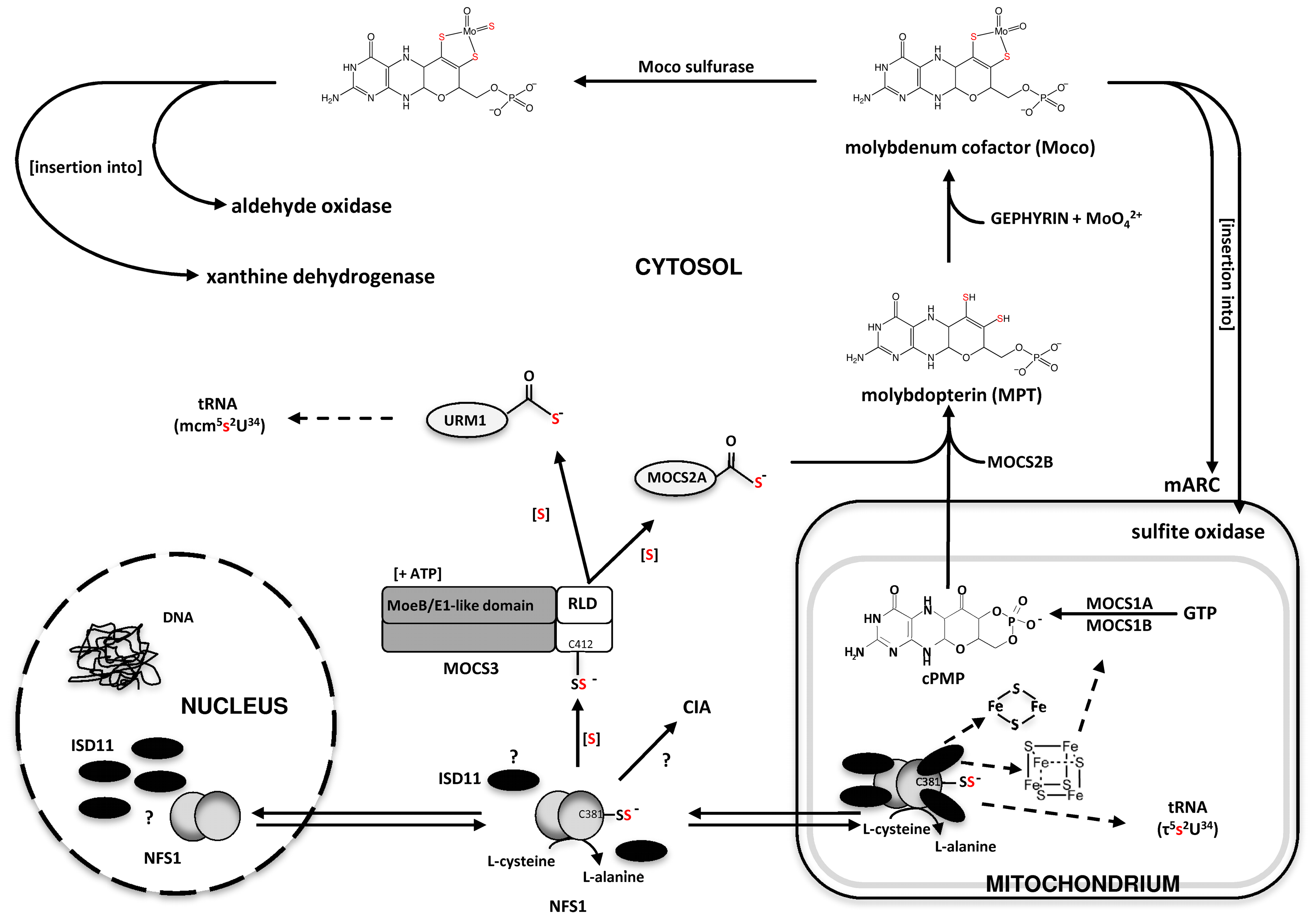

Supplement: Figure S4 — Model for Moco biosynthesis and FeS cluster biosynthesis in the cell. NFS1 and ISD11 are predominately targeted to the mitochondria but additionally were detected in the nucleus. In the mitochondria, the NFS1/ISD11 complex is the sulfur donor for FeS cluster biogenesis and for the thiomodification of mitochondrial tRNAs. In the cytosol, NFS1 interacts with the rhodanese-like domain (RLD) of MOCS3 transferring its protein-bound persulfide-sulfur from NFS1-Cys381 to MOCS3-RLD-Cys412. MOCS3 adenylates MOCS2A and URM1 by its N-terminal MoeB/E1-like domain and further forms a thiocarboxylate on both proteins by sulfur transfer from the C-terminal RLD. URM1 is involved in the thiolation of the wobble base thiouridine to 5-methoxycarbonylmethyl-2-thiouridine (mcm5s2U34) in cytoplasmic tRNAs while MOCS2A forms with MOCS2B the active MPT synthase and transfers the sulfur for the formation of MPT in Moco biosynthesis. Moco is important for the activity of the molybdoenzymes sulfite oxidase, mARC, xanthine dehydrogenase and aldehyde oxidase. The function of NFS1 and ISD11 in the nucleus remains unknown. It is also not clear whether ISD11 has a role in the cytosol. (TIF) [file pone.0060869.s004.tif]
